# Supplementary material for: ProteinShader: illustrative rendering of macromolecules
Source: BMC Struct Biol. 2009 Mar 30;9:19. doi: 10.1186/1472-6807-9-19 (PMC2672931; doi:10.1186/1472-6807-9-19)
Supplement: Additional file 1 — ProteinShader program without source code. This compressed file contains the complete ProteinShader program including associated libraries, but no source code. A README.txt file gives an overview of the ProteinShader distribution, and the index.html file in the help subdirectory has directions on getting started with the program as well as a set of tutorials. [file 1472-6807-9-19-S1.zip › ProteinShader-beta-0_9_4-binary/help/api/org/proteinshader/structure/visitor/package-summary.html]

org.proteinshader.structure.visitor (ProteinShader API)


|  |  |  |
| --- | --- | --- |
| **Overview** \| **Package** \| Class \| **Use** \| **Tree** \| **Deprecated** \| **Index** \| **Help** | |  |
| **PREV PACKAGE**   **NEXT PACKAGE** | **FRAMES**    **NO FRAMES**     **All Classes** |  |


---

## Package org.proteinshader.structure.visitor

Holds Visitor classes that know how to traverse the hierarchy of objects
held by class Structure.

**See:**
  
          **Description**

| **Interface Summary** | |
| --- | --- |
| **Visitable** | This interface requires an accept( Visitor ) method, which should be used to call back to the Visitor so that the Visitor can perform one or more operations on the Visitable object. |

| **Class Summary** | |
| --- | --- |
| **AminoAcidLabelVisitor** | Traverses the AminoAcids of a Structure and builds a non-redundant list of residueIDs. |
| **BondDestroyerVisitor** | Removes Bonds on all Atoms that it visits. |
| **BondGeneratorVisitor** | Generates standard Bonds for AminoAcids and Waters. |
| **BondPredictor** | Knows how to test if the distance between two Atoms is acceptable for a covalent Bond. |
| **BoundsVisitor** | Knows how to find the min and max xyz-coordinate values for all Atoms in each Model. |
| **FrenetFrameGeneratorVisitor** | Calculates a discrete Frenet frame for each AminoAcid visited. |
| **LoopGeneratorVisitor** | Generates Loop objects from any AminoAcids of a Chain that do not already belong to a Helix or BetaStrand. |
| **ModifierVisitor** | Knows how to modify multiple Drawable objects while traversing the hierarchy of objects contained in a Structure. |
| **SFWriterVisitor** | A simple file writer subclass of Visitor that writes to a file all of the data from each Model in a Structure. |
| **VisibilityVisitor** | Knows how to traverse the Structure object hierarchy and find all visible Drawable objects. |
| **Visitor** | This self-propelled Visitor knows how to traverse the hierarchy of data objects contained by a Structure. |

## Package org.proteinshader.structure.visitor Description

Holds Visitor classes that know how to traverse the hierarchy of objects
held by class Structure.
